# A BIM-GIS Framework Integrated with CCTV Analytics for Urban Walkability Assessment

**DOI:** 10.3390/s25123637

**Published:** 2025-06-10

**Authors:** Mingzhu Wang, Peter Kok-Yiu Wong, Jack C. P. Cheng

**Affiliations:** 1 Department of Architecture and Civil Engineering, City University of Hong Kong, Hong Kong 999077, China; 2 Department of Civil and Environmental Engineering, The Hong Kong University of Science and Technology, Hong Kong 999077, China

**Keywords:** BIM-GIS integration, CCTV analytics, computer vision, real-time crowd monitoring, walkability assessment, data schema

## Abstract

This study proposes a novel framework integrating Building Information Modeling (BIM) and Geographic Information Systems (GIS) with real-time crowd analytics from Closed-Circuit Television (CCTV) for quantitative walkability assessment. The framework extends open data standards (IFC and CityGML) to model infrastructural and pedestrian flow attributes comprehensively. A walkability scoring mechanism quantifies route quality based on accessibility, efficiency, and physical comfort, differentiating among pedestrian groups, such as individuals sensitive to weather conditions or carrying belongings. Implemented at the Hong Kong University of Science and Technology (HKUST), results indicate that the framework effectively captures variations in walkability scores due to directional differences (uphill vs. downhill), crowd conditions, and operational constraints like facility closures. Statistical tests confirm significant differences in walking costs across these scenarios with variations of up to 30%, demonstrating the framework’s robustness and practical utility for real-time, human-centric urban infrastructure planning.

## 1. Introduction

Urbanization is accelerating worldwide, with the United Nations projecting that 68% of the global population will live in urban areas by 2050 [1]. This trend significantly increases in-town travel demand, highlighting walking as a vital mode of mobility. Walking contributes not only to environmental sustainability and public health [2,3], but also to economic vibrancy, land value, and urban vitality [4,5,6]. Recognizing these benefits, many cities have prioritized walkability in their transport and urban planning strategies, such as the 3rd Comprehensive Transport Study (CTS-3) in Hong Kong [7], strategic studies for walkability improvement in central areas by different governments including Singapore [8], New York City [9] and London [10].

Despite growing interest, effective walkability monitoring remains challenging due to two key issues: multidimensional nature and regional uniqueness. Walkability encompasses both objective infrastructural features, such as path connectivity and barrier-free access, and subjective experiences like comfort, aesthetics, and weather protection [11]. Moreover, walkability conditions vary by local topography and demographics, influencing trip purposes and route choices [12,13]. These complexities demand a comprehensive, human-centric framework that accounts for diverse spatial and behavioral variables.

Traditional walkability assessment methods such as statutory design guidelines, pedestrian audits and surveys, aim to analyze the region-specific characteristics of existing infrastructural conditions and pedestrian walking behaviors. Nevertheless, these approaches are time-consuming, resource-intensive and lack scalability. Digital models of the as-built environment is a promising solution, which can capture the overall topography and spatial relationship among different facilities for walkability evaluation. Specifically, Geographic Information System (GIS) provides topographic data and network analysis between POIs. Building Information Modeling (BIM) capture fine-grained topology information such as volumetric spaces, and semantic attributes such as air-conditioning along certain facilities [14]. However, existing models rarely integrate these technologies into a cohesive framework for walkability evaluation, nor do they adequately incorporate real-time human movement data.

Crowd dynamics, particularly pedestrian congestion, play a crucial role in perceived and actual walkability. Integrating real-time behavioral data with spatial models can evaluate the influence of pedestrians on the walkability of different paths, enhancing navigation and decision-making. Video analytics is promising for recognizing and analyzing pedestrians walking trajectories and physical attributes from closed-circuit television (CCTV) cameras. For example, Yin et al. [15] and Chen et al. [16] estimated the pedestrian volumes of various street segments from street view images available online. Chmielewska et al. [17] monitored pedestrian density in a carpark to determine busy-hours in a day. However, existing CCTV-based approaches primarily focus on pedestrian counting or basic density estimation, neglecting dynamic aspects like real-time congestion impacts on route choice. Market solutions such as Hikvision’s crowd density analytics and Axis Communications’ pedestrian flow monitoring offer real-time counting but lack comprehensive integration with spatial infrastructure data, limiting their utility for detailed urban planning tasks. Thus, there remains a significant gap concerning the integration of real-time crowd dynamics with detailed spatial modeling.

To address these gaps, this study proposes a BIM-GIS framework enhanced by CCTV-based crowd analytics for comprehensive walkability evaluation. More specifically, a walkability evaluation framework is proposed to formalize the walkability-related information requirement, integrating infrastructural characteristics modeled with BIM and GIS, and operational crowd flow condition using CCTV analytics. The framework extends openBIM (IFC) and openGIS (CityGML) schemas to capture topological, geometric, and semantic attributes within a 3D walkability network. A walkability scoring mechanism is developed to quantify route costs based on accessibility, walking efficiency, and physical comfort, tailored to different pedestrian groups and dynamic environmental conditions. This human-centric framework supports responsive navigation, scenario analysis, and inclusive infrastructure design, offering a scalable tool for walkability evaluation in urban environments. The contributions are as follows:

(1) Extension of openBIM (IFC) and openGIS (CityGML) schemas to capture walkability-relevant attributes comprehensively.
(2) Integration of real-time pedestrian flow data from CCTV-based crowd analytics into BIM-GIS infrastructure models for dynamic walkability mapping.
(3) Development of a walkability scoring mechanism accounting for accessibility, efficiency, and comfort, tailored to diverse pedestrian conditions and environmental contexts.

## 2. Literature Review

### 2.1. BIM-GIS for Modeling Infrastructural Attributes

BIM-GIS integration has been widely explored to digitize the spatial and semantic characteristics of built environments for walkability analysis. A key challenge lies in the time-consuming process of manually constructing high-detail 3D models. Therefore, researchers have proposed defining essential geometric and semantic properties to guide model reconstruction. Leslie et al. [18] utilized GIS databases to identify environmental characteristics such as dwelling density, road connectivity, land-use and zoning data, correlated to walking behavior. However, their analysis remained at a coarse areal scale, lacking fine-grained semantic details at the streetscape level. Lin [19] examined visual obstructions using GIS-based line-of-sight analysis but overlooked topographic elements like slope gradients, which notably affect walkability for vulnerable groups. Several studies have focused on extending data schemas to improve accessibility modeling. Park et al. [20] extended the IndoorGML schema to capture mobility-assistive facilities, enabling 3D navigation for disabled users in indoor environments. Shin and Lee [21] extracted spatial properties from BIM model for path analyses, in terms of path length, no. of turns, space volumes and connectivity. However, many of these efforts emphasize geometric over semantic properties, omitting experiential factors such as comfort or path preference. Yin [19] and others noted the lack of attention to directional differences in walking effort, which can significantly influence route selection. Moreover, these studies mainly extract spatial properties of the walking paths and spaces, ignoring their semantic properties that also affect pedestrian comfort and hence path selection.

Other works have explored more semantic-rich representations. Hamieh et al. [22] proposed a graph model based on Industry Foundation Classes (IFC) that supports accessibility-aware navigation within buildings. Although effective for finding shortest paths, the model did not consider walking comfort or environmental conditions. Wheeler et al. [23] extended CityGML to include semantic attributes of pathways, including weatherproof and lighting conditions that affect pedestrian comfort when walking. Yet, they did not include dynamic attributes, e.g., crowdedness. Li et al. [24] introduced the concept of representing crowds in BIM as “blob spaces” to reflect discomfort due to visibility loss and movement disruption. However, their model treated crowds as static elements, ignoring temporal and spatial variations in pedestrian density.

Collectively, these studies highlight the potential of BIM and GIS to support walkability evaluation through detailed infrastructure modeling. Nevertheless, most existing approaches rely on static attributes and lack integration with real-time operational data such as crowd movement. Furthermore, few have formalized walkability attributes within standardized open data schemas like IFC and CityGML, limiting data interoperability and scalability. Addressing these gaps requires a more holistic framework that integrates dynamic behavioral data with comprehensive infrastructural modeling to enable more precise, context-aware walkability analyses.

### 2.2. Walkability Scoring Mechanisms for Analyses

Numerous studies have sought to define and quantify walkability through various environmental and experiential indicators. Ewing and Handy [25] identified five urban design qualities, such as visual enclosure and streetscape variation, derived from expert-rated features like street furniture, signage, and pedestrian activity. Liu and Jiang [26] focused on accessibility to essential urban facilities (e.g., markets, schools, transport) using origin-destination modeling based on travel distance. However, both studies emphasized macro-scale transitions between POIs and overlooked the detailed attributes of the walking paths themselves. To address mobility diversity, Park et al. [20] modeled indoor navigation networks for disabled individuals, highlighting route differences due to facility availability (e.g., elevators vs. escalators).

Zhou et al. [27] quantified visual walkability using enclosure, greenery, paving, and crowding indicators. Similarly, Shin and Lee [21] assessed design quality based on distance, connectivity, simplicity, and spatial volume. Apart from geometric attributes like the quality of sidewalk pavements, Arellana et al. [28] emphasized subjective comfort and aesthetic perception, incorporating survey-based evaluations into walkability maps. Zhang et al. [29] quantified various attributes from BIM to assess elderly friendliness based on lighting, barrier-free features, and guardrails. Gao et al. [30] and Yang et al. [31] considered sidewalk safety, noise, and pedestrian flow variation by age or trip purpose. Despite these advances, most models remain static and fail to incorporate environmental dynamics such as real-time crowding.

Furthermore, walkability scores often assume homogeneity across pedestrian groups, neglecting the distinct needs of the elderly, disabled, or weather-sensitive individuals. Some efforts, like Yin [19], integrated field-based pedestrian density into walkability scores, interpreting higher volumes as indicators of popularity. However, such interpretations may overlook the negative impact of excessive crowding on comfort and safety. Li et al. [32] proposed a more nuanced approach by correlating walkability with pedestrian density, noise, and light levels—factors linked to user comfort—but relied on labor-intensive field measurements, limiting scalability and real-time applicability.

Overall, while existing literature has contributed valuable frameworks, there remains a need for a dynamic and group-sensitive walkability scoring mechanism. Such a system should incorporate time-varying operational conditions (e.g., crowd levels, route disruptions) and personalize evaluations based on diverse user profiles to support more realistic, human-centric walkability assessments.

### 2.3. Walkability Assessment and Urban Analytics

In recent years, computer vision and deep learning have been increasingly employed to assess walkability through street-level imagery. Many studies utilize platforms like Google Street View (GSV), Baidu, or Tencent to extract visual features such as sidewalk presence, greenery, building facades, and street width. For example, Li et al. [32,33] applied CNNs to panoramic street images to evaluate the coverage of greenery and vehicular road space, integrating virtual reality to capture subjective walkability perceptions. Ogawa et al. [34] further demonstrated the predictive capability of CNNs in inferring perceived cleanliness, spaciousness, and attractiveness directly from GSV. Similarly, Blečić et al. [35] classified street segments into five walkability levels based on CNN analysis and visualized the results for regional evaluation.

Several researchers have focused on quantifying walkability using interpretable indicators derived from image elements. For example, Zhou et al. [27] analyzed Baidu Map imagery using CNNs to develop walkability indices based on features like enclosure and greenery. Yin and Wang [36] assessed visual enclosure by measuring sky occlusion from tall buildings and linked these metrics to pedestrian flow. While these methods effectively capture spatial characteristics, many are trained on crowdsourced labels, which introduces potential bias, and their models often function as black boxes—offering limited interpretability or validation.

In parallel, video analytics has been applied to surveillance footage to extract dynamic pedestrian behaviors, particularly for behavior analysis and safety monitoring. Early work by Willis et al. [12] and Ismail et al. [37] extracted walking trajectories and pedestrian–vehicle interactions from video to study crowd behavior and collision risk. More recent studies by Zhang et al. [38] and Cheng et al. [39] used video analytics in enclosed environments (e.g., metro stations, campuses) to estimate crowd density and facilitate emergency evacuation planning. Yin et al. [15] and Chen et al. [16] analyzed pedestrian volumes citywide using time-stamped street view imagery, providing large-scale visibility into pedestrian behavior during peak hours. However, these approaches often focus on safety or operational efficiency rather than holistic walkability experiences.

To enrich image-based methods, Li et al. [40] and Kang et al. [41] proposed integrating environmental sensors and GIS data with deep learning for multicriteria evaluation, considering noise, lighting, and air quality. Huang et al. [42] advanced this further by combining street-level imagery with big data analytics to generate multi-scale walkability maps that reflect both spatial and behavioral dynamics. Collectively, these works signal a shift toward more comprehensive and adaptive walkability assessments.

Growing attention has also been paid to group-specific walkability, especially towards vulnerable populations. Liu et al. [43] introduced a deep learning-based detection pipeline that identifies older pedestrians and aging-unfriendly infrastructure, enabling targeted evaluation of age-inclusive pedestrian environment. Jeon and Woo [44] used street panorama imagery and CNNs to examine how walkability varies across socioeconomic groups. These efforts align with Huang et al. [45], who quantified how specific physical features, like facade transparency, tree canopy, and commercial density, influence visual walkability perception, using image classification models and survey data.

Nonetheless, existing studies predominantly rely on static imagery or context-specific applications like evacuation. There remains a lack of integration between real-time human dynamics and spatial infrastructure models. Furthermore, few studies consider inclusive mobility, overlooking the varied mobility needs of children, older adults, and persons with disabilities. A more holistic framework is needed, which defines walkability in both spatial and temporal terms and supports personalized evaluation across diverse pedestrian groups.

## 3. Materials and Methods

**Figure 1:**
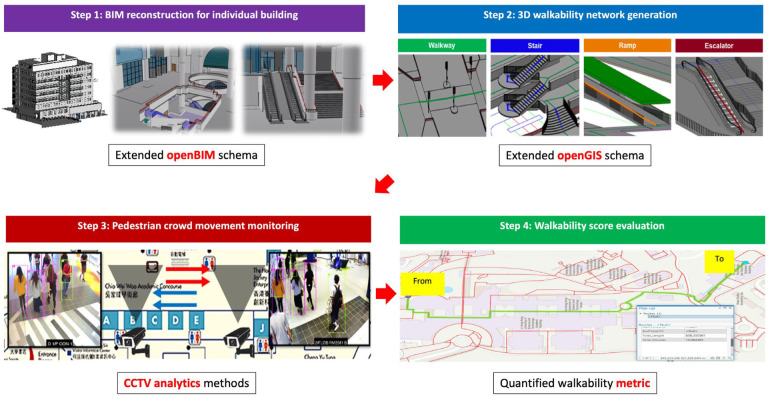
Proposed framework for quantitative walkability evaluation.

This paper develops a framework that integrates the infrastructural characteristics of walking facilities with pedestrian flow data to support both walkability evaluation and pedestrian navigation, as illustrated in Figure 1. The overall methodology comprises four main steps. Steps 1 and 2 involve extending openBIM and openGIS schemas to model individual buildings and construct a walkability network, respectively. These extensions, based on open data standards, ensure interoperability and facilitate integration across platforms and throughout the infrastructure lifecycle. Together, they formalize the walkability-related information requirements. Step 3 incorporates real-time pedestrian flow data, extracted through CCTV analytics, as dynamic attributes within the walkability network. Finally, Step 4 introduces a walkability scoring mechanism that quantifies walking costs based on accessibility, efficiency, and comfort, enabling comprehensive and data-driven walkability assessment.

### 3.1. Extended IFC Schema for Modeling Individual Buildings in openBIM

**Figure 2:**
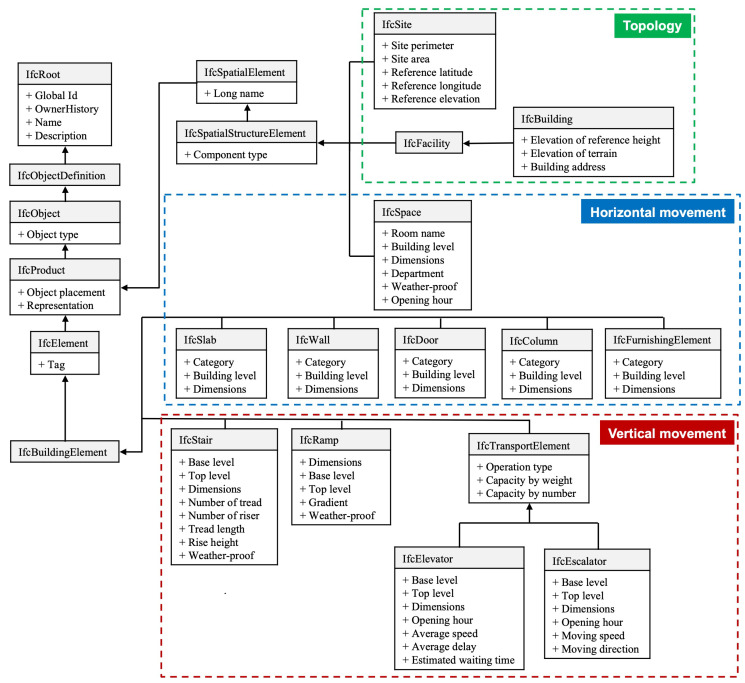
Extended IFC data schema for modeling individual building in openBIM.

To support walkability evaluation, the required information of built environments is formalized using open data standards. Specifically, the openBIM framework is extended to govern the BIM reconstruction process, enabling the capture of both 3D geometry and semantic attributes relevant to pedestrian movement. Figure 2 illustrates the extended IFC schema developed to represent walkability-related features of individual buildings. The proposed schema categorizes data into three key domains: (1) topological information for geo-referencing, (2) horizontal movement facilities, and (3) vertical movement facilities.

(1) Topological information. The *IfcSite* entity stores the geographic location of a building with reference to latitude, longitude, and elevation, and the *IfcBuilding* entity stores the terrain elevation and address of the building, enabling accurate geo-referencing of building models in GIS platforms. This ensures spatial alignment when integrating multiple BIM models within a larger urban terrain.
(2) Horizontal movement. The *IfcBuildingElement* entity encompasses the list of facility components inside a building that pedestrians walk along or pass through. The *IfcBuildingElement* category includes components that define walkable spaces and obstacles within buildings. *IfcSlab* represents floors and ceilings, while *IfcWall* defines spatial boundaries. *IfcDoor* models transitions between different sub-areas, and *IfcColumn* and *IfcFurnishingElement* represent structural or furniture-related obstructions that may reduce effective walkway width. These elements are linked to attributes such as dimensions and building levels, which help extract usable floor space. *IfcSpace* further defines rooms or corridors as walkable units, with attributes like weather protection and opening hours to support time-sensitive walkability analysis.
(3) Vertical movement. Vertical connectivity is crucial for inclusive walkability. The schema includes *IfcStair* (modeled with riser count and height), *IfcRamp* (with width and gradient), and *IfcTransportElement* for elevators and escalators. *IfcElevator* captures capacity, opening hours, average speed, delay, and estimated waiting time. *IfcEscalator* includes speed, operational direction, and schedule. All vertical elements store information on connected floor levels, enabling analysis of inter-floor accessibility for users with different mobility needs.

By modeling buildings using this extended IFC schema, all infrastructure-related attributes necessary for walkability analysis are encoded within the BIM environment. This structured data is subsequently integrated into GIS in the next phase to construct a spatially enabled digital twin for walkability assessment.

### 3.2. Extended CityGML Schema for Modeling Walkability Network in openGIS

**Figure 3:**
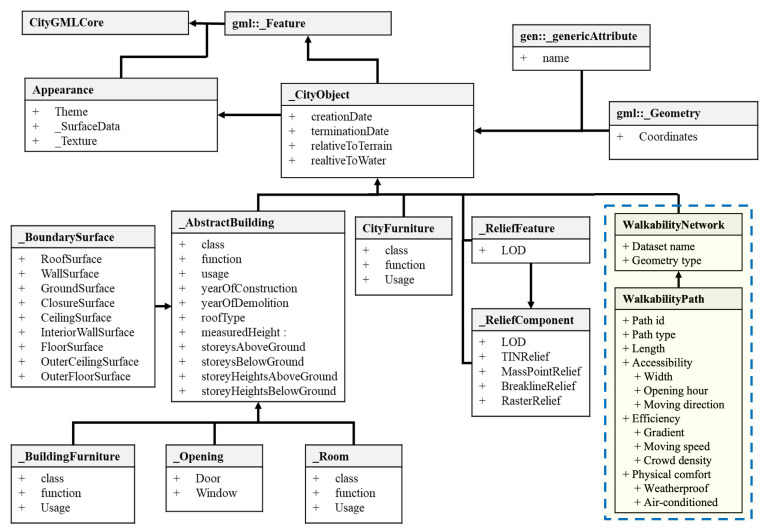
Extended CityGML data schema for modeling walkability network in openGIS. The dotted box indicates the entities created for walkability network and path.

Following the modeling of individual buildings using the extended IFC schema, these BIM models are integrated into a GIS environment to form a unified digital twin. This integration aims to preserve both the geometric and semantic attributes defined in BIM while enabling spatial alignment across multiple sub-models. The walkability network is then constructed by extracting relevant spatial, semantic, and topological information from this integrated digital twin. Figure 3 presents the extended CityGML schema that governs the structure of the walkability network.

**Figure 4:**
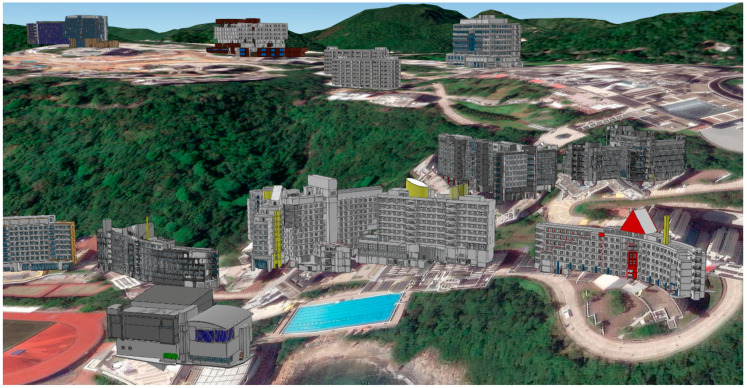
Illustration of multiple BIM models geo-referenced in GIS environment.

To represent the 3D topological context, a digital terrain model is incorporated into the GIS platform, capturing elevation variations across roads and hillsides. Each BIM building model is geo-referenced on this terrain using coordinates and elevation data stored in *IfcSite* and *IfcBuilding*. Figure 4 illustrates the result of such integration, where each building is accurately placed within the terrain. Importantly, semantic attributes encoded in BIM—such as room functions, furniture elements, or opening hours—are preserved through schema mapping.

Importantly, the semantic attributes of each building stored in BIM are preserved after integrating with GIS. Our framework realizes efficient integration between BIM and GIS through developing interoperable data models based on open standards, i.e., IFC and CityGML. By developing data schemas, the information is interoperable across different platforms and hence, the modeled elements and attributes can be mapped. For example, the *IfcSpace* entity in the IFC can be mapped to *_Room* entity in CityGML to represent individual room or space, *IfcDoor* can be mapped to *_Opening* to represent door elements and *IfcFurnishingElement* can be mapped to *_BuildingFurniture* to represent furniture elements inside buildings. As a result, a comprehensive BIM-GIS digital twin is formed, which serves as the basis for walkability network construction, by extracting the walkable area and associated attributes. This approach streamlines data import, alignment, and integration processes within GIS platforms, significantly reducing manual intervention and enhancing consistency.

**Figure 5:**
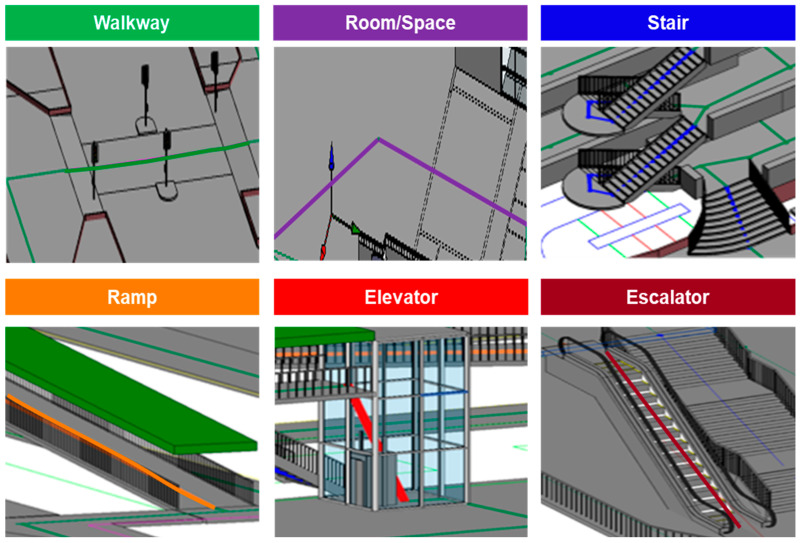
Illustration of generated walkability paths along different facilities.

The walkability network itself is represented by a series of interconnected paths abstracted from walkable spaces in the digital twin. As defined in the extended CityGML schema (Figure 3), the *WalkabilityNetwork* entity stores such a network with basic attributes like its name and geometry type, and consists of multiple *WalkabilityPath* entities that represent individual walkable segments. Each path is defined as a line or polyline that mimics the walking path of a person along certain facility components. The construction of the walking network is based on the BIM-GIS model created, which captures the infrastructural layout to assist in modeling the facility type and connectivity of each path. As illustrated in Figure 5, six types of paths are categorized. This study adopts the path generation method proposed by Zhang and Chiaradia [46], which models centerlines for walkways or spaces and connects them based on BIM-GIS-defined spatial relationships. Horizontal paths represent indoor and outdoor walkable areas, while inclined polylines represent vertical connections such as stairs, ramps, escalators, and elevators. The vertical connectivity—i.e., the start and end floor levels of each vertical facility—is extracted directly from the integrated BIM-GIS data.

Beyond geometry, each path is assigned semantic attributes to support walkability evaluation. As summarized in Table 1, these attributes are grouped under three core walkability dimensions: (1) accessibility, (2) efficiency, and (3) physical comfort. These annotated paths form a complete walkability network, enabling both spatial analysis and user-specific walkability scoring in the later stages of the framework.

(1) *Accessibility*. Accessibility defines whether a path is usable by a particular pedestrian group. Path width, for instance, may restrict access for individuals carrying bulky items or wheelchair users, particularly in narrow corridors or ramps. Time-dependent availability is also captured through opening hours—for example, elevators may be unavailable outside designated service times. Directionality is another constraint; facilities such as escalators may operate in only one direction, making reverse travel infeasible. These factors are encoded to support user-specific route filtering based on physical and temporal constraints.
(2) *Efficiency*. Efficiency refers to attributes that influence movement speed and travel time. Path length is the basic determinant of travel duration. Gradient plays a critical role—uphill paths or steep ramps can significantly reduce walking speed. For mechanically assisted paths, the moving speed of elevators and escalators is modeled, along with elevator delay factors such as door opening/closing time and expected wait time. Additionally, real-time crowd density, which will be derived from CCTV analytics as described in Section 3.3, is incorporated. Higher density, especially with counter-directional flow, can reduce walkability by slowing down pedestrian movement and increasing perceived effort.
(3) *Physical comfort*. This category captures environmental and perceptual factors that influence route preference, even when travel time is unaffected. Lack of weather protection (e.g., covered walkways) may discourage use during adverse weather conditions such as rain or extreme heat. Air-conditioning is another key factor, especially in warm climates, where pedestrians often prefer indoor, climate-controlled routes over sun-exposed or poorly ventilated alternatives.

By incorporating these attributes, the extended data schema enables the construction of a detailed walkability network that combines both static infrastructural elements from the digital twin model and dynamic operational data from CCTV analytics. This dual integration supports more informed path-finding and personalized walkability evaluations across diverse user needs and environmental conditions.

### 3.3. Real-Time Crowd Monitoring with CCTV Analytics

As introduced in Section 3.2, pedestrian walking efficiency along a path is influenced by real-time crowd conditions. To account for this, the proposed framework incorporates an automated CCTV video analytics module for real-time crowd density estimation, leveraging the pedestrian tracking and attribute recognition system developed in our previous work [47]. The framework can detect and track individual persons accurately despite practical challenging conditions, e.g., occlusion, appearance ambiguity and lighting variability, obtaining accurate people count and individual walking trajectory in a region. Specifically, the system combines YOLOv5 for person detection with DeepSORT-based tracking, and integrates visual, motion, and high-level features (e.g., gender, age, belongings) for robust re-identification. A similarity measure integrating multiple cues enhances identity matching. Also, a probation mechanism is incorporated to mitigate the influence of occlusions and environmental changes such as lighting variability. In our extensive experiments, the system achieves a MOTA of 88.4% and IDF1 of 90.3% across diverse indoor and outdoor scenes. Even under moderate occlusion, the probation mechanism boosts tracking performance, validating its reliability for pedestrian density estimation. Bounding boxes generated from tracking are used to compute both pedestrian count and motion trajectories for each monitored region.

**Figure 6:**
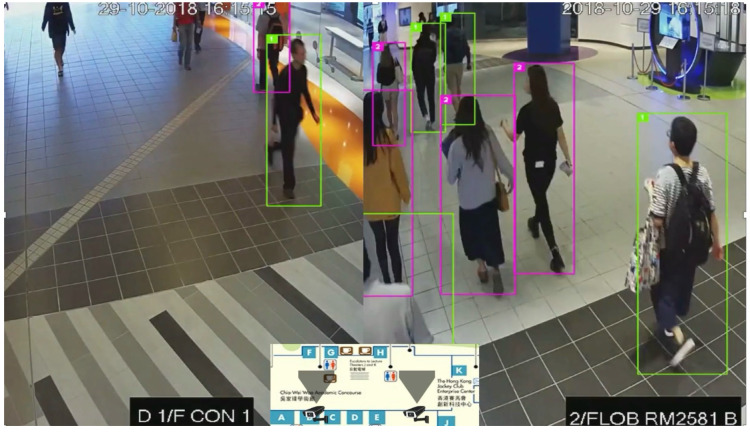
Illustration of CCTV processing results for real-time crowd monitoring. Non-English terms are the Chinese translations of the places in the map.

As shown in Figure 6, the system identifies each person as a bounding box, continuously tracking positions over time and producing anonymized outputs that include people count and movement trajectories. Based on the CCTV processing output, the crowd movement in each area can be analyzed and incorporated into walkability monitoring. To mitigate privacy and ethical issues, the framework implements anonymization techniques by design. Specifically, the video analytics pipeline uses real-time object detection and tracking algorithms that extract only anonymous positional and trajectory data without storing identifiable visual information. While CCTV coverage forms the primary sensing modality, the framework can be extended with other sensors (e.g., infrared counters, Bluetooth, or Wi-Fi probes) to enhance robustness in areas lacking full visual coverage.

**Figure 7:**
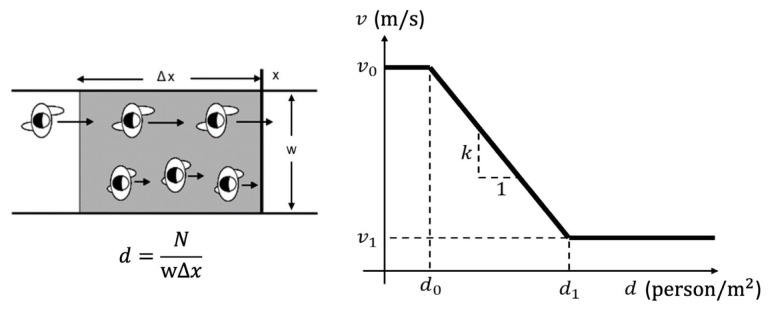
Methodology of speed–density relationship modeling (based on Cheng et al. [39]).

To incorporate crowd conditions into the walkability network, a three-step methodology is employed:

(1) Mapping camera views to walkability paths. Each CCTV camera’s view is modeled as a polygon, which is spatially matched with overlapping path segments in the walkability network. This allows crowd data from each camera to be directly associated with the relevant path(s). If a path segment falls within a camera’s field of view, its identifier is linked to that segment, enabling spatiotemporal aggregation of crowd conditions across the network.
(2) Calibrating speed–density relationships. To quantify the influence of crowding on walking speed, a speed–density relationship is calibrated using CCTV outputs. Following the approach by Cheng et al. [39], a step function is used to model the relationship due to its simplicity and empirical validity. For each path segment, crowd density is calculated as the number of people divided by the segment’s area (length × width). Simultaneously, individual walking speeds are extracted from trajectory data. These speed–density pairs are used to generate a calibration curve representing typical pedestrian flow behavior under varying densities (see Figure 7).
(3) Predicting walking speed from real-time density. During real-time operation, the calibrated speed–density curve is used to infer walking speeds for each path segment based on current crowd density. These predicted speeds are then assigned to the corresponding paths in the walkability network. Section 3.4 will further explain how this speed information contributes to path scoring and pedestrian route recommendations.

### 3.4. Proposed Mechanism of Walkability Scoring for Path Finding

Based on the constructed walkability network, a walkability scoring mechanism is developed to quantify the walking cost along a path. The proposed cost function will be the basis of finding a least-cost path, which enables comparison across alternative routes, supporting identification of suboptimal designs and facilitating walkability analysis and improvement. The walkability scoring mechanism is defined by two equations. Equation (1) computes the walking cost $C_{p}$ for an individual path segment *p*, while Equation (2) calculates the total cost $C_{route}$ for a route composed of multiple segments:

(1) $$C_{p}={a_{p}}^{-1}\times \left(\frac{l_{p}}{v_{p}}+t_{delay}^{p}+t_{wait}^{p}\right)\times f_{weather}^{p}$$

(2) $$C_{route}=\sum_{p}C_{p}$$

where

- $a_{p}$: Accessibility of a path segment, either 0 (inaccessible) or 1 (accessible);
- $l_{p}$: Length of a path segment;
- $v_{p}$: Walking/moving speed;
- $t_{delay}^{p}$: Time of delay (e.g., elevator operation);
- $t_{wait}^{p}$: Time of waiting (e.g., elevator queue);
- $f_{weather}^{p}$: Weather-related cost multiplier.

The accessibility variable $a_{p}$ accounts for physical and operational constraints, including path width, barrier-free design elements (e.g., ramps vs. stairs), and opening hours, which may vary across pedestrian groups. Paths are deemed inaccessible (i.e., $a_{p}$ = 0) if they violate any constraint for the target user profile, thereby being excluded from viable route options. Walking speed $v_{p}$ is influenced by gradient, walking direction (e.g., uphill vs. downhill), real-time crowd density, and pedestrian group characteristics (e.g., elderly or burdened users). For mechanical facilities such as elevators and escalators, $v_{p}$ corresponds to their operational speed. $t_{delay}^{p}$ captures expected operational delays along the path, such as door opening/closing cycles for elevators, while $t_{wait}^{p}$ reflects estimated queueing time, which is modeled as a function of real-time user load (from CCTV analytics) and facility capacity. These variables are parametrized based on service schedules and empirical delay measurements collected during field observation. The expression $\left(\frac{l_{p}}{v_{p}}+t_{delay}^{p}+t_{wait}^{p}\right)$ computes the total walking or travel time along the segment. This is multiplied by the inverse of accessibility ${a_{p}}^{-1}\;$ and further adjusted by a physical comfort factor $f_{weather}^{p}$. The weather-related multiplier accounts for environmental discomfort in adverse conditions, such as exposure to heat or rain on non-weatherproof paths, or preference for air-conditioned indoor facilities.

**Figure 8:**
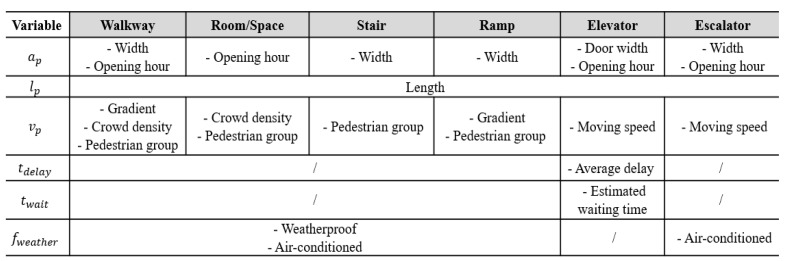
Walkability attributes involved in defining each variable for each type of facility.

Figure 8 summarizes the walkability attributes contributing to each variable in the cost function, categorized by facility type. Weighting factors for each component were calibrated through empirical observation, expert consultation, and pedestrian surveys conducted at HKUST, ensuring context-sensitive scoring reflective of real-world conditions. While the current weighting factors were empirically derived via expert consultation and campus pedestrian surveys, the scoring function design is modular and extensible. Future implementation can incorporate data-driven calibration techniques, such as Bayesian optimization and genetic algorithms, to fine-tune parameters based on observed user route choices or walking times. This would improve generalizability in other cities or demographic contexts.

## 4. Experiments and Results

### 4.1. Experimental Design

To validate the proposed BIM-GIS framework, a real-world case study was conducted at The Hong Kong University of Science and Technology (HKUST). The HKUST campus was selected not only due to data availability, but because it represents a complex pedestrian environment, including multi-level buildings, varying topography, indoor-outdoor path transitions, and dynamically changing pedestrian flow conditions. The selected Points of Interest (POIs) span diverse walking scenarios, such as uphill/downhill travel, barrier-free access requirements, and exposure to weather-sensitive segments, enabling a realistic simulation of different pedestrian constraints. The project is part of a broader initiative to develop a digital twin of the entire HKUST campus, aiming to digitize its physical infrastructure and assets for sustainable, smart campus management. The campus’ geometry and topology are reconstructed into a unified digital platform, serving as a single source of truth to support walkability analysis and informed infrastructure planning.

#### 4.1.1. Construction of Digital Twin Database with BIM-GIS

BIM models of over 60 campus buildings were modeled in Autodesk Revit based on the extended IFC schema introduced in Section 3.1. The models include detailed geometric and semantic attributes, with accuracy verified through 3D laser scanning to support precise walkability assessments. These BIM models were then geo-referenced and integrated into a digital terrain model covering approximately 500,000 m^2^ using Esri ArcGIS Pro v3.0. The integration was performed under open data standards to ensure full interoperability and preservation of attribute data.

**Figure 9:**
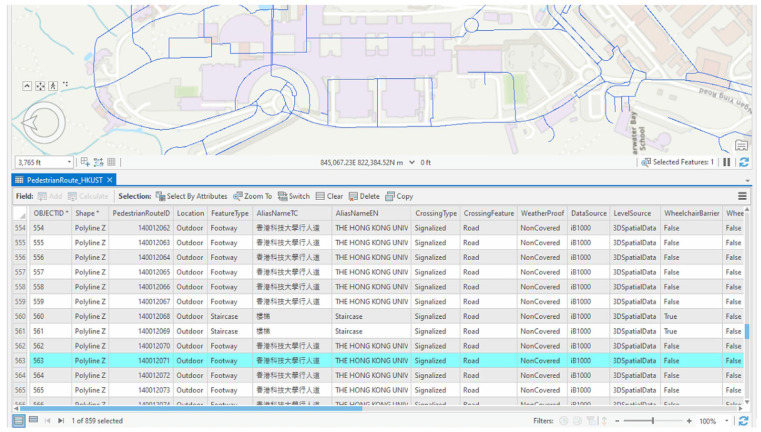
Original 3D walking network obtained from Hong Kong government data store. The non-English terms represent the Chinese name of the infrastructure.

**Figure 10:**
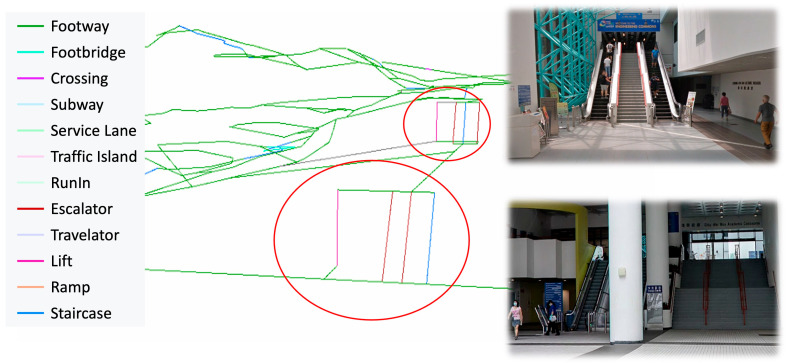
Enriched walkability network categorizing across-floor facilities in indoor areas. The red circles indicate the location of excavators as shown in the captured image.

To extract walkable areas for path analyses, the 3D pedestrian walking network created by the Lands Department and Transport Department was incorporated. As illustrated in Figure 9, this dataset includes 3D polylines annotated with attributes such as facility type, weatherproof coverage, and gradient. The 3D network was further enriched with walkability-related attributes according to the extended CityGML schema described in Section 3.2. Additional vertical circulation elements—lifts, escalators, and staircases—were embedded into the network to reflect indoor level transitions within academic buildings (Figure 10). Customized walkability scoring functions, developed in Section 3.4, were configured and applied using ArcGIS Network Analyst to evaluate route costs and validate the proposed walkability scoring mechanism.

#### 4.1.2. Selected POIs for Walkability Analyses

**Figure 11:**
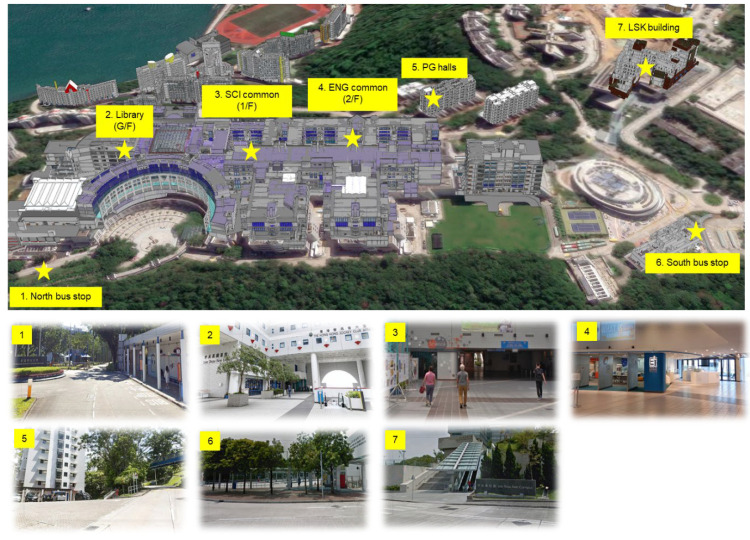
List of POIs selected for walkability analyses in HKUST.

To support realistic and meaningful walkability evaluation, seven key Points of Interest (POIs) within the HKUST campus were selected. These locations represent areas frequently visited or traversed by students and staff as part of their daily routines. As shown in Figure 11, these POIs also capture a variety of spatial and environmental features, such as elevation changes and outdoor exposure, that influence walkability across different user profiles. The selected POIs include:

- POIs 1 and 6: The North and South bus stops, serving as main access points to the campus via public transportation.
- POIs 2–4: The Library (G/F), the School of Science common space (1/F), and the School of Engineering common space (2/F), which are high-traffic indoor areas with distinct vertical separations.
- POI 5: The postgraduate (PG) residential halls, located along a long outdoor slope, introducing weather exposure and increased physical exertion.
- POI 7: The Lee Shau Kee (LSK) Business Building, situated on elevated terrain, requiring extended travel uphill from other campus zones.

These POIs enable the comparative analysis of walkability under varied physical and environmental conditions, particularly when examined across different pedestrian groups with distinct mobility needs.

#### 4.1.3. Categorization of Pedestrian Groups and Movement Behaviors

**Figure 12:**
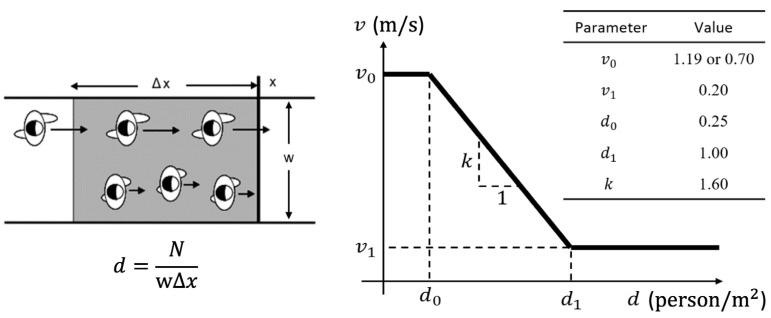
Speed–density relationship defined regarding the crowdedness of a path.

To better evaluate walkability under diverse user conditions, three pedestrian groups were defined, each characterized by distinct movement behaviors and physical constraints. This categorization supports differentiated walkability scoring and reflects real-world variability in pedestrian needs. The group definitions are informed by prior studies [48,49], with key physical parameters summarized in Table 2, Table 3 and Table 4, and Figure 12.

(1) Normal persons. This group represents pedestrians without specific mobility constraints. Their movement is primarily affected by distance and environmental factors such as crowd density. Walkability scores for this group serve as a baseline in comparative analysis.
(2) Weather-sensitive persons. Individuals in this group are more affected by adverse weather conditions (e.g., rain, strong sun). Outdoor paths without weatherproofing impose greater perceived discomfort and thus higher walking costs. These individuals may prefer longer but indoor or sheltered routes, as quantified by discomfort multipliers shown in Table 4.
(3) Belonging-carried persons. This category includes delivery personnel, elderly pedestrians using walking aids, and individuals with physical disabilities. Their movement is restricted on certain facilities, such as narrow ramps or staircases, and their walking speed is significantly reduced. In some cases, specific facilities (e.g., escalators or steep slopes) may be considered non-accessible for this group.

In addition, Figure 12 presents the walking speed adjustments based on crowd density, following the speed–density relationship outlined in Section 3.3. Compared to the baseline proposed by Cheng et al. [39], which was based on emergency evacuation scenarios, this study adopts more conservative initial walking speeds (1.19 m/s for normal persons and 0.70 m/s for belonging-carried persons) to reflect typical pedestrian behavior under normal conditions.

### 4.2. Comparing Walkability Scores Among Pedestrian Groups

To demonstrate the effectiveness of the proposed walkability metric in capturing diverse mobility needs, walkability scores were analyzed for three pedestrian groups: (1) normal persons, (2) belonging-carried persons, and (3) weather-sensitive persons. Table 5 presents the estimated walking costs (in minutes) between selected POIs introduced in Section 4.1.2 for each group. These scores were validated against field-surveyed walking times and perceived physical discomfort, confirming their realism and practical interpretability.

**Figure 13:**
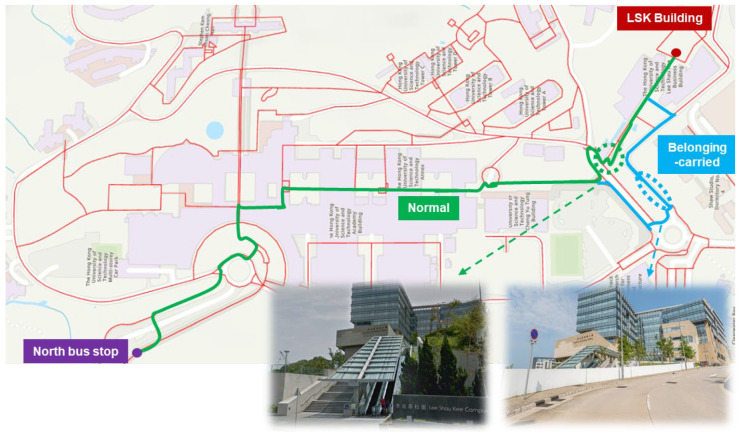
Recommended detouring for belonging-carried persons towards LSK building.

Comparative analysis reveals that both the belonging-carried and weather-sensitive groups experience noticeably higher walking costs, particularly for routes involving elevation change or outdoor exposure. For example, the route from the North Bus Stop to the LSK building takes 18.4 min for belonging-carried persons, a 30.5% increase compared to 14.1 min for normal persons. This increase is attributed to the lack of accessible vertical transportation options such as elevators. As shown in Figure 13, only stairs and escalators are available along the direct (green) route, which are inaccessible to individuals with mobility limitations. Consequently, a detour along the sloped (blue) path is recommended, significantly increasing both distance and effort. The addition of auxiliary vertical movement facilities, such as elevators near this route, would improve accessibility for this group.

**Figure 14:**
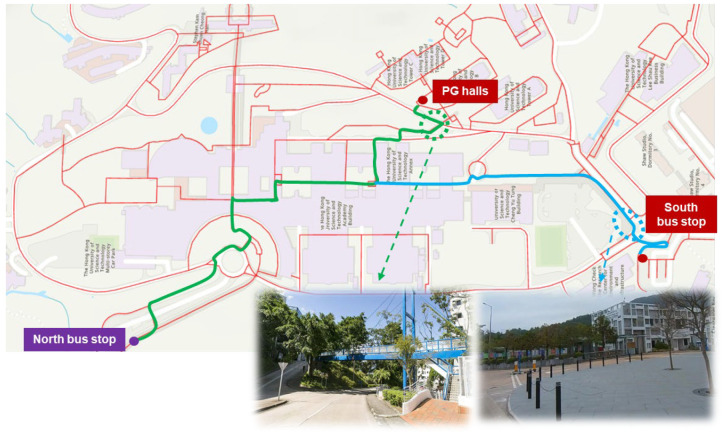
Recommended routes for weather-sensitive persons towards two POIs.

For weather-sensitive pedestrians, walking costs increase notably for routes exposed to outdoor conditions. The travel time to the PG halls increases by 22.4% (13.1 vs. 10.7 min), and the travel time to the South Bus Stop increases by 18.3% (12.9 vs. 10.9 min), relative to normal persons. These paths lack overhead coverage, leading to increased discomfort in adverse weather. As illustrated in Figure 14, large segments of these routes are uncovered, and no fully sheltered alternatives are currently available. Incorporating weatherproof infrastructure, such as covered walkways or canopies, along these paths would substantially improve walkability for this group.

To validate that the walkability scores vary significantly across different pedestrian profiles, we applied the Friedman test to the six origin-destination pairs presented in Table 5. The results indicate statistically significant differences in walkability scores among the normal, belonging-carried, and weather-sensitive groups (χ^2^ = 9.09, *p* = 0.011), confirming that the framework is sensitive to diverse pedestrian needs.

Overall, the results confirm that the proposed walkability metric effectively captures the impact of accessibility, environmental exposure, and mobility constraints on pedestrian experience. This enables data-driven recommendations for infrastructure improvements tailored to the needs of diverse user groups.

### 4.3. Comparing Walkability Scores Considering Directional Difference

This section evaluates how the proposed walkability metric captures directional differences, particularly the increased effort required for uphill travel, by comparing walking costs for the same routes in both uphill and downhill directions. The analysis focuses solely on the normal pedestrian group to isolate the effect of elevation changes without additional behavioral or physical constraints. Table 6 present the walkability scores (in minutes) for downhill and uphill movement between selected POIs and the percentage increase in walking costs for uphill travel relative to downhill.

**Figure 15:**
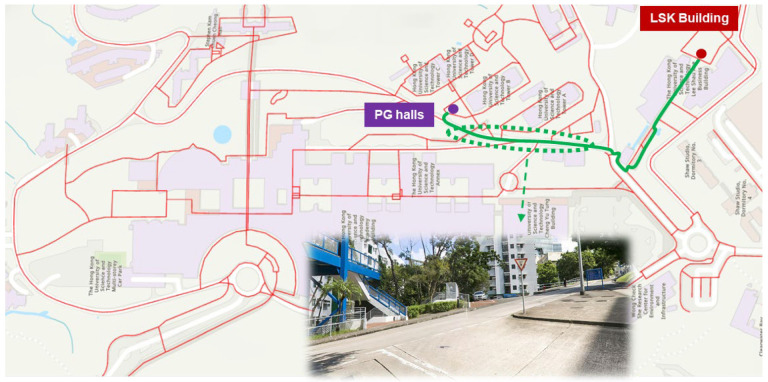
Illustration of the long outdoor slope between PG halls and LSK building.

Overall, uphill routes result in 6–16% higher walking costs compared to their downhill counterparts. The route from PG Halls to the LSK building shows the largest increase (16%), attributed to the extended outdoor slope with an average gradient of 0.15, as illustrated in Figure 15. This emphasizes the importance of modeling slopes and elevation when computing pedestrian effort in real-world environments.

**Figure 16:**
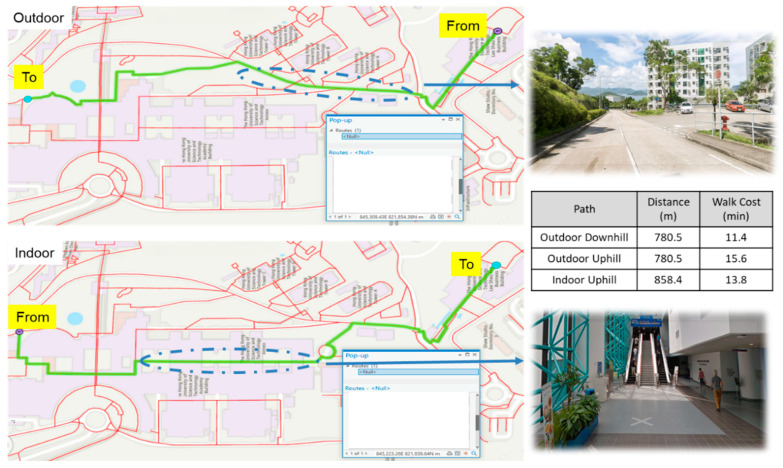
Different walkability scores for the same path due to directional difference.

The proposed walkability framework incorporates such directional asymmetry into its path-finding logic. Figure 16 presents two scenarios for a normal person walking between the same pair of locations in opposite directions. In the downhill scenario (upper figure), the shortest route along the outdoor slope is recommended as the least-cost path, since distance dominates cost. In contrast, the uphill scenario (lower figure) results in a different recommendation: an indoor path with escalator access is preferred over the shorter outdoor slope (858.4 m vs. 780.5 m), as the walking cost is lower (13.8 vs. 15.6 min), which is a 13% improvement. This example highlights the system’s ability to account for elevation-induced differences and recommend direction-sensitive paths accordingly. By modeling topographic attributes and mobility effort asymmetrically, the framework supports more realistic and user-centered pedestrian navigation.

A Wilcoxon signed-rank test was conducted to compare walking costs between uphill and downhill routes across six matched paths (Table 6). The test yielded a statistically significant difference (Z = 0.0, *p* = 0.031), confirming that the proposed scoring framework appropriately accounts for the additional effort associated with uphill movement.

### 4.4. Comparing Walkability Scores Considering Operational Incidents

**Figure 17:**
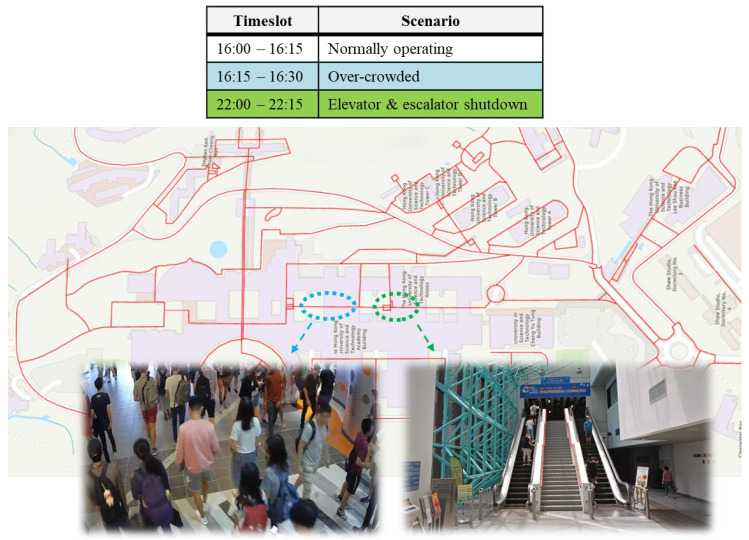
A schedule of different scenarios happening at different timeslots.

This section evaluates how the proposed walkability metric captures time-dependent operational factors, specifically crowd congestion and facility shutdowns, that influence pedestrian movement. These scenarios reflect real campus conditions at different times of day, as shown in Figure 17. Two key timeslots are analyzed:

- 16:15–16:30 (Class transition period): A peak pedestrian flow window between class sessions, leading to increased crowd density within academic buildings.
- After 22:00 (Late evening): Several elevators and escalators are shut down, potentially affecting route accessibility for mobility-constrained users such as the belonging-carried group.

Table 7 consolidates walkability scores for the belonging-carried group under three scenarios: (1) normal, (2) over-crowded, and (3) facility shutdown.

**Figure 18:**
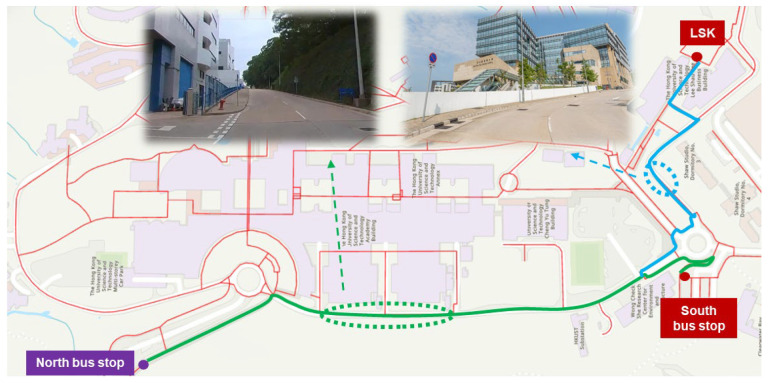
Recommended detouring for avoiding an over-crowded condition.

During the class transition period, routes passing through the academic building (e.g., to SCI Common, ENG Common, and PG Halls) exhibit noticeable cost increases due to discomfort from crowd density. For example, walking costs to SCI Common increase by 10.3%, to ENG Common by 14.3%, and to PG Halls by 16.2%, reflecting the system’s ability to quantify discomfort-induced delays. Additionally, routes to the South Bus Stop and LSK also incur increases of 14.7% and 12.5%, respectively. These are not directly due to discomfort but result from the system recommending detours to avoid crowded indoor paths. As shown in Figure 18, the system reroutes pedestrians via outdoor slopes, where increased walking effort, especially uphill, contributes to higher costs.

**Figure 19:**
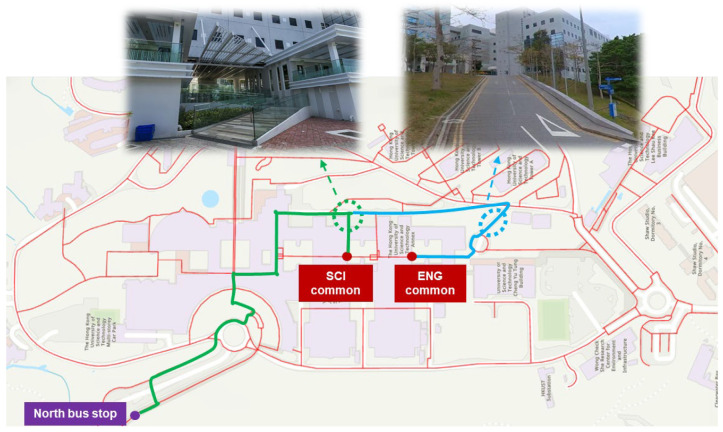
Recommended detouring due to shutdown of elevators and escalators.

In the late evening scenario (after 22:00), shutdown of elevators and escalators further increases walking costs for mobility-limited users. For example, walking to SCI Common increases by 23.0% (10.7 vs. 8.7 min) and to ENG Common by 33.7% (13.1 vs. 9.8 min). Since stairs become the only available vertical movement option within academic buildings, and are avoided by the belonging-carried group, these routes are excluded. The system instead recommends longer, outdoor detours with ramp access, as illustrated in Figure 19. The extended detour and elevation gain further increase effort, particularly for reaching ENG Common. Interestingly, routes to South Bus Stop and LSK remain unchanged between the over-crowded and shutdown scenarios. In both cases, the indoor facilities are avoided due to either discomfort or inaccessibility, resulting in the same least-cost paths being recommended.

To assess the impact of time-dependent operational changes, a Friedman test was performed on the walkability scores of the belonging-carried group across normal, over-crowded, and facility shutdown scenarios (Table 7). The test revealed a statistically significant difference (χ^2^ = 9.33, *p* = 0.009), supporting the model’s sensitivity to real-time infrastructure conditions and user accessibility.

These findings validate that the proposed walkability framework effectively incorporates time-sensitive operational dynamics, such as crowd density and facility availability, into pedestrian path evaluation. By dynamically adjusting route recommendations based on real-time and scheduled conditions, the system supports more informed, human-centric pedestrian navigation.

## 5. Discussion

The proposed BIM-GIS-integrated framework provides a robust and dynamic approach for walkability evaluation, combining static infrastructural features with real-time crowd analytics. By fusing detailed building models and geospatial context with surveillance-based behavioral data, the framework supports more comprehensive and human-centric assessments of urban mobility.

The experimental results validate the effectiveness of the proposed framework in capturing context-sensitive walkability across diverse pedestrian groups, routes, and environmental conditions. The walkability scoring system successfully reflected differences in accessibility, comfort, and efficiency, accounting for variations in pedestrian mobility (e.g., belonging-carried, weather-sensitive), walking direction (uphill vs. downhill), and operational incidents (e.g., crowd congestion, facility shutdowns). These findings demonstrate the system’s ability to generate realistic and actionable insights into pedestrian experience and infrastructure performance.

Although the study was conducted within the HKUST campus, the framework is designed with extensibility in mind. The use of open data standards, specifically IFC for BIM and CityGML for GIS, ensures interoperability and adaptability across various urban contexts. Furthermore, the modular architecture of the system allows for flexible calibration of walkability parameters according to local infrastructure, behavioral norms, and climatic factors. This adaptability supports deployment in other dense urban areas, university campuses, business parks, or transportation hubs. However, to generalize the system effectively, practitioners must consider variations in available infrastructure (especially CCTV coverage), local pedestrian behavior profiles, and terrain complexity. In areas where surveillance infrastructure is limited, integrating complementary sensing modalities (e.g., Wi-Fi-based tracking or mobile phone location data) could enhance robustness.

Despite its strengths, the framework has several limitations. First, its effectiveness depends on the availability and strategic placement of CCTV infrastructure, which may be limited in some urban areas. In such cases, incomplete coverage can lead to gaps in crowd data. This could be addressed through optimized camera placement or integration with alternative sensing methods such as infrared counters, Bluetooth, or Wi-Fi probes. Second, while the walkability scoring system is empirically calibrated for the HKUST context, generalizing it to other urban environments may require additional local data and expert input. Walking preferences vary across demographics and cultures, and scoring parameters may need to be adjusted accordingly. Third, the integration of BIM and GIS, though based on standardized schemas, remains resource-intensive and requires technical expertise. Compared to simpler GIS-only or survey-based approaches, the framework demands higher initial investment in data modeling and system deployment.

Future work may explore integrating simulation tools (e.g., agent-based modeling) to predict pedestrian flow under hypothetical scenarios, such as infrastructure modifications or weather events. Additionally, incorporating machine learning models to dynamically adjust scoring functions based on observed pedestrian behaviors over time could further improve adaptability. Broader evaluations across different city types and population groups will also be crucial in validating the framework’s generalizability and informing guidelines for standardized walkability modeling.

## 6. Conclusions and Future Work

This paper presents a comprehensive framework that integrates infrastructural modeling with pedestrian behavior analytics to support quantitative walkability evaluation. The framework extends openBIM (IFC) and openGIS (CityGML) standards to formally capture both geometric and semantic attributes relevant to walkability, enabling the construction of a 3D walkability network. In addition to static infrastructural elements, the framework incorporates dynamic operational conditions such as real-time pedestrian crowdedness obtained through CCTV analytics. A walkability scoring mechanism is developed to quantify route quality in terms of accessibility, walking efficiency, and physical comfort. The scoring model accounts for individual pedestrian characteristics (e.g., weather sensitivity, load carrying) and contextual variations (e.g., walking direction and facility status), supporting personalized and context-aware analyses. The framework is validated through a case study at the Hong Kong University of Science and Technology (HKUST), where statistically significant differences in walking costs were observed across pedestrian groups, directional conditions, and operational scenarios, reinforcing the framework’s sensitivity and practical reliability.

The key research contributions of this study are as follows:

- A unified BIM-GIS-based framework for walkability evaluation that formalizes spatial and semantic data requirements and integrates real-time crowd conditions.
- Extension of the IFC schema to represent walkability attributes within individual buildings, including support for both horizontal and vertical pedestrian movement.
- Extension of the CityGML schema to support seamless integration of detailed building models into georeferenced terrain for network-level walkability assessment.
- A robust scoring mechanism that reflects the varying walking costs experienced by different user groups under diverse environmental conditions.

While CCTV-based pedestrian flow estimation enhances the dynamic aspect of the model, it is limited by spatial coverage and sensitivity to environmental factors like lighting and occlusion. Future research may explore the integration of alternative sensing modalities, such as infrared sensors, Bluetooth, Wi-Fi fingerprinting, or GPS tracking, and the application of adaptive multimodal data fusion to further improve the reliability and scalability of human-centric walkability monitoring systems.

## Figures and Tables

**Table 1:** Six types of paths and corresponding walkability attributes to be modeled.

Walkway	Room/Space	Stair	Ramp	Elevator	Escalator
Width	Opening hour	Width	Width	Door width	Width
Opening hour	Length	Length	Length	Opening hour	Opening hour
Length	Crowd density	Weatherproof	Gradient	Length	Moving direction
Gradient	Weatherproof	Air-conditioned	Weatherproof	Moving speed	Length
Crowd density	Air-conditioned		Air-conditioned	Average delay	Moving speed
Weatherproof				Estimated waiting time	Air-conditioned
Air-conditioned					

**Table 2:** Walking speeds of different pedestrian groups (in meters/second).

Facility\Group	Normal	Weather-Sensitive	Belonging-Carried
Ordinary walkways	1.190		0.700
Stairs (upwards)	0.682		/
Stairs (downwards)	0.776		/

**Table 3:** Slope-dependent walking speeds of different pedestrian groups (in meters/second).

Slope\Group	Normal/Weather-Sensitive		Belonging-Carried	
Degree	Upwards	Downwards	Upwards	Downwards
0.000	1.190	1.190	0.700	0.700
2.860	1.146	1.243	0.674	0.731
5.710	1.066	1.252	0.627	0.737
8.530	0.977	1.261	0.575	0.742

**Table 4:** Discomfort factors due to adverse weather for different pedestrian groups.

Weather\Group	Normal	Weather-Sensitive	Belonging-Carried
Cloudy (normal)	1.00	1.00	
Sunny	1.03	1.15	
Rainy	1.05	1.35	

**Table 5:** Walkability scores among selected POIs for three groups of pedestrians (minutes).

From (North Bus Stop)	Library	SCI Common	ENG Common	PG Halls	South Bus Stop	LSK
Normal	4.8	8.1	9.3	10.7	10.9	14.1
Belonging-carried	4.8	8.7	9.8	11.7	10.9	18.4
Weather-sensitive	5.4	9.4	10.1	13.1	12.9	15.6

**Table 6:** Walkability scores of walking downhill and uphill among selected POIs (in minutes).

From (LSK)	South Bus Stop	PG Halls	ENG Common	SCI Common	Library	North Bus Stop
Walk downhill	4.8	5.0	5.7	7.4	9.1	13.3
Walk uphill	5.3	5.8	6.3	8.0	9.8	14.1
Difference	10.4%	16%	10.5%	8.1%	7.7%	6.0%

**Table 7:** Walkability scores (minutes) for belonging-carried persons under different scenarios.

From (North Bus Stop)	Library	SCI Common	ENG Common	PG Halls	South Bus Stop	LSK
Normal	4.8	8.7	9.8	11.7	10.9	18.4
Over-crowded	4.8	9.6	11.2	13.6	12.5	20.7
Facility shutdown	4.8	10.7	13.1	14.2	12.5	20.7

## Data Availability

The datasets presented in this article are not readily available because the data are part of an ongoing project.
